# Does the effect of disability acquisition on mental health differ by employment characteristics? A longitudinal fixed-effects analysis

**DOI:** 10.1007/s00127-019-01783-x

**Published:** 2019-10-24

**Authors:** Zoe Aitken, Julie Anne Simpson, Rebecca Bentley, Allison Milner, Anthony Daniel LaMontagne, Anne Marie Kavanagh

**Affiliations:** 1 Disability and Health Unit, Centre for Health Equity, Melbourne School of Population and Global Health, The University of Melbourne, 207 Bouverie Street, 3010 Carlton, VIC Australia; 2 Biostatistics Unit, Centre for Epidemiology and Biostatistics, Melbourne School of Population and Global Health, The University of Melbourne, 207 Bouverie Street, 3010 Carlton, VIC Australia; 3 Gender and Women’s Health Unit, Centre for Health Equity, Melbourne School of Population and Global Health, The University of Melbourne, 207 Bouverie Street, 3010 Carlton, VIC Australia; 4 Centre for Population Health Research, School of Health and Social Development, Deakin University, Melbourne Burwood Campus, 221 Burwood Highway, 3125 Burwood, VIC Australia

**Keywords:** Mental health, Disability, Social epidemiology, Health inequalities, Employment, Effect modification

## Abstract

**Purpose:**

Longitudinal studies have suggested a causal relationship between disability acquisition and mental health, but there is substantial heterogeneity in the magnitude of the effect. Previous studies have provided evidence that socioeconomic characteristics can buffer the effect but have not examined the role of employment characteristics.

**Methods:**

We used data from 17 annual waves of the Household, Income and Labour Dynamics in Australia Survey to compare the mental health of working age individuals before and after disability acquisition, using the Mental Health Inventory, a subscale of the SF-36 health questionnaire. Linear fixed-effects regression models were used to estimate the effect of disability acquisition on mental health. We tested for effect modification by two characteristics of people’s employment prior to disability acquisition: occupational skill level and contract type. Multiple imputation using chained equations was used to handle missing data.

**Results:**

Disability acquisition was associated with a substantial decline in mental health score (estimated mean difference: − 4.3, 95% CI − 5.0, − 3.5). There was evidence of effect modification by occupational skill level, with the largest effects seen for those in low-skilled jobs (− 6.1, 95% CI − 7.6, − 4.5), but not for contract type.

**Conclusions:**

The findings highlight the need for social and health policies that focus on increasing employment rates, improving the sustainability of employment, and providing employment services and education and training opportunities for people who acquire a disability, particularly for people in low-skilled occupations, to reduce the mental health inequalities experienced by people with disabilities.

**Electronic supplementary material:**

The online version of this article (10.1007/s00127-019-01783-x) contains supplementary material, which is available to authorized users.

## Introduction

Globally, it is estimated that 15% of the population currently live with a disability [1]. People with disabilities experience large mental health inequalities compared to those without disability [2–4]. There is evidence from longitudinal studies that disability acquisition leads to deterioration in people’s mental health, suggesting the existence of a causal relationship between disability and poor mental health [5–12]. Notably, there is a great deal of heterogeneity in the magnitude of this effect; some people experience substantial mental health declines whereas others experience little or no decline [13].

According to the International Classification of Functioning, Disability and Health (ICF) framework, disability results from the interaction between people’s health conditions, individual characteristics and social factors [14]. Conceived in this way, social factors and socioeconomic inequalities are likely to affect how a health condition or impairment impacts on people’s functioning and restrictions to participation in society. This highlights the importance of examining the interaction between disability and social factors when examining mental health effects associated with disability acquisition.

Previous research has suggested that socioeconomic disadvantage exacerbates the negative effect of disability acquisition on mental health. Six longitudinal studies have examined whether the association between disability acquisition and mental health differs according to people’s socioeconomic characteristics prior to disability, and found evidence that the association varied by income [6], housing characteristics [7], social support [5], relationship status [6], education [9], and wealth [8, 10]. Importantly, these results suggest that favourable socioeconomic circumstances may provide a buffer against the detrimental effect of disability on mental health. The evidence about how employment characteristics affect the relationship between disability and mental health is sparse. An Australian study found larger negative effects for people who were unemployed prior to disability, though the study did not have the statistical power to detect an interaction [6].

This is an important gap in the literature. Evidence about the psychosocial benefits of employment on mental health in the general population, such as the positive impacts of high psychosocial job quality on mental health [15, 16] and the negative effects associated with transitions from employment to unemployment [17], have led us to hypothesize that characteristics of people’s employment may attenuate the effect of disability on people’s mental health. Casual or temporary employment has been hypothesised to adversely affect mental health [18], but previous studies in the Australian general working population have been null [19]. Nevertheless, casual employment—due to its lack of security, paid sick leave, and other adverse working conditions—could be more important for people with disability and could modify the disability acquisition–mental health relationship. A better understanding of the effects of employment characteristics may identify subgroups of people who are particularly vulnerable to large mental health declines associated with disability acquisition and may give insight into the mechanisms linking disability and mental health [20], which could inform the development of targeted social and health policies.

This analysis uses data from a large Australian longitudinal study to model relationships between disability acquisition and mental health in working age individuals, testing how employment characteristics and disability acquisition combine to influence mental health. We test for effect modification by employment characteristics prior to disability acquisition using two characteristics of people’s employment, occupational skill level and contract type, to quantify excess mental health effects of disability acquisition associated with these characteristics.

## Methods

### Data source

We used data from 17 waves of the Household, Income and Labour Dynamics in Australia (HILDA) Survey, a nationally representative longitudinal study of Australian households and individuals which collects information annually on a wide range of social, demographic, health and economic characteristics [21]. The original panel was selected in 2001 and included 13,969 individuals from 7682 households, sampled using a national probability sample of private dwellings. In subsequent waves, survey members included all original participants, household members attaining the age of 15 years, and new participants when existing participants formed new households. A top up sample was added in 2011 to maintain representativeness. At each wave, data were collected on each household member, and face-to-face interviews were sought from all household members aged 15 years or above. The response rate for participation in the survey was 80% and attrition between waves was about 6%. We used data from individuals aged 25 to 64 years to represent the working age population.

### Mental health

Mental health was measured in every wave using the mental health inventory (MHI), one of the eight subscales of the Short Form 36 (SF-36) health questionnaire. The SF-36 is a widely used self-completion measure of health status that has been validated for use in the Australian population and to detect within-person changes in health over time [22]. The mental health subscale assesses symptoms of anxiety, depression and positive aspects of mental health. It has been shown to be psychometrically sound [23], and an effective screening tool for mood and anxiety disorders and severe depressive symptomatology [24–31]. It includes five questions relating to mental health over the previous 4 weeks, each scored using five response categories, which are summed and rescaled into a continuous measure ranging from 0 to 100. Higher scores represent better mental health.

### Disability acquisition

Information on disabilities was collected in every wave. Participants were asked if they had an “impairment, long-term health condition or disability which restricts their everyday activities that had lasted, or was likely to last, for a period of 6 months or more”. Participants were defined as having acquired a disability if they reported, in consecutive years, two waves with no disability followed immediately by two waves with a disability. A minimum of two consecutive waves was used so as to identify people with relatively longer-term disability [32–34]. All consecutive waves in which individuals reported a disability subsequent to disability acquisition were also included in analyses as well as all consecutive waves reporting no disability prior to disability acquisition (minimum of four, maximum of 17 consecutive waves contributed for each person). If participants reported more than one episode of disability acquisition, only the first episode was included.

### Employment characteristics

Occupational skill level was defined using the Australian and New Zealand Standard Classification of Occupations, a skill-based classification of occupations. Information on occupation was combined into a measure with three mutually exclusive categories: high skill jobs (managers; professionals); medium skill jobs (technicians and trades workers; community and personal service workers; clerical and administrative workers); and low skill jobs (sales workers, machinery operators and drivers, labourers). Contract type was categorised as permanent (ongoing) employment; fixed-term contract; self-employed (employee of one’s own business or self-employed); and casual (in a casual or temporary contract or employed through a labour-hire firm or temporary employment agency). For each variable, we generated response categories to additionally specify people who were not in the labour force (not actively seeking employment, for various reasons including education, retirement, infirmity/disability, or household duties) and unemployed (actively seeking employment or unable to find work in the last 4 weeks). To represent employment characteristics prior to disability acquisition, variables were recorded as time-invariant, measured two waves prior to the first year of reported disability.

### Missing data

We used multiple imputation (MI) using chained equations with 50 imputations to maximise the validity of findings (note < 0.1% missing data for disability so these individuals were excluded). This approach assumes that the missing data were missing at random, that the systematic differences in the distribution of missing and observed variables are explained by differences in observed data [35]. The imputation model included all variables in the fixed-effects analysis as well as additional auxiliary variables including self-rated health, education, household income, relationship status and children. To account for potentially important interactions in the analysis model (i.e. ensure congeniality between the analysis and imputation model), we imputed missing values separately for waves with and without disability to account for interactions [36].

### Statistical analysis

The characteristics of the sample with complete data were described at baseline (participants’ first wave contributing to the sample) and employment characteristics were summarised two waves prior to the first year of reported disability. Mean mental health scores for waves with and without disability were compared across categories of employment characteristics. For each individual, mean mental health was calculated for waves in which they reported a disability and waves in which they reported no disability (within-person mean mental health), and these were then pooled to summarise the mean mental health scores for the sample (between-persons).

**Fig. 1:**
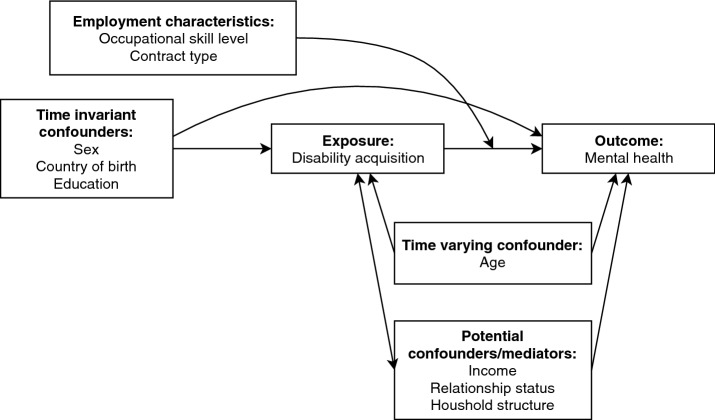
Causal diagram illustrating postulated causal relationships between disability acquisition and mental health

We used fixed-effects longitudinal linear regression models to estimate the association between disability acquisition and mental health score, using the imputed data. Regression coefficients from the models describe within-person estimated mean differences in mental health scores between waves in which they reported a disability and waves in which they reported no disability. Fixed-effects models estimate changes in outcomes associated with changes in exposure status within individuals, rather than between individuals, therefore controlling for individual-level factors that do vary over time. In this way, the models remove bias from time-invariant confounding from both measured and unobserved variables [37]. Covariates were included in regression models if they were deemed to be potential time-varying confounders, i.e., common causes of both disability acquisition and mental health. We considered a single covariate, age measured as a continuous variable (see Fig. 1). Though income, household structure and relationship status could be conceived as potential confounders, they were not included as covariates in models as they were likely to be affected by disability acquisition and therefore be potential mediators of the association. Including mediators as covariates in the models would bias the effect estimates by adjusting for variables on the causal pathway between the exposure and outcome.

To assess whether the association between disability acquisition and mental health varied by prior employment characteristics, we included an interaction term between disability acquisition and employment characteristics, with separate models for each employment characteristic. We assessed whether there was statistical evidence of effect modification on the additive scale using Wald tests assessing whether the interaction term coefficients were different to zero. Analyses were conducted in Stata/SE 15 [38], using the mi estimate and xtreg commands with fixed-effects estimators and robust standard errors to fit regression models. A complete case analysis on the sample of people with complete data on all variables was carried out as a comparison to the primary analysis using MI.

### Sensitivity analysis

We conducted two sensitivity analyses to test the robustness of our findings. Firstly, we excluded people acquiring disability relating to a psychological impairment as the mental health effect and the influence of employment characteristics may differ for this subgroup. Secondly, we restricted the waves of data contributing to the sample to the two waves following disability acquisition, because some people may have only two waves of data prior or post disability, whereas other may have up to 15 and the fixed-effects models assume that a contemporaneous effect that does not change over time.

## Results

**Fig. 2:**
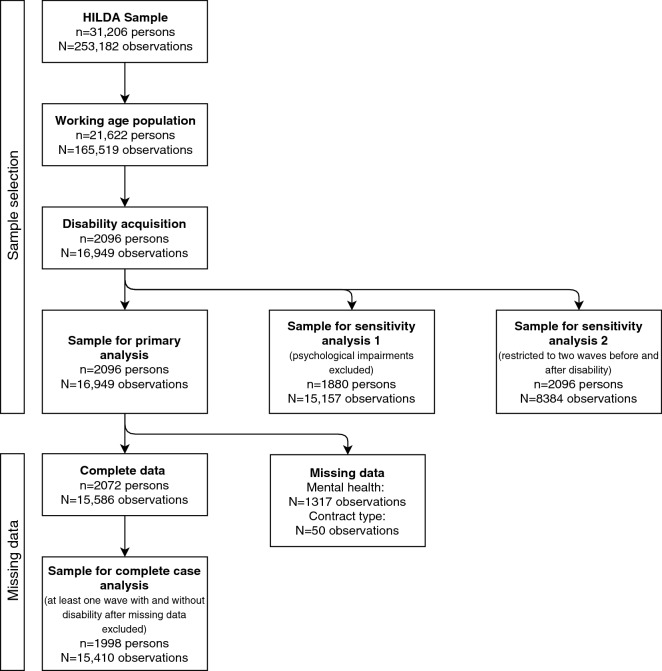
Flow diagram illustration selection of eligible sample and details of missing data

Across the 17 waves of data, there were 2096 individuals of working age who met our definition of disability acquisition contributing a total of 16,949 observations, with a mean number of eight observations per person (four preceding disability and four subsequent to disability on average). Complete data were available for 2072 people and 15,586 observations, with missing data for 8% of observations (Fig. 2). Of these, 1998 people had at least one wave of data in which they reported no disability and one wave of data in which they did report a disability (15,410 observations), and therefore contributed to the sample for the complete case analysis.

Table S1 compares the characteristics of observations with complete data versus missing data (see Online Resource). Missing observations were more common for people who were younger, male, born outside of Australia, not in a relationship, with poorer mental health, and those who experienced socioeconomic disadvantage, including low education, low income and low skilled jobs.

### Descriptive analyses

At baseline, the mean age of participants in the sample was 42 years, 54.5% were women, 78.9% were born in Australia and 31.9% had not completed secondary school education (Table 1). Prior to disability, 29.1% were in high-skilled jobs, 30.5% were in medium-skilled jobs, and 18.6% in low-skilled jobs; 46.9% were in permanent employment, 5.5% on fixed-term contracts, 14.2% self-employed, and 11.7% in casual employment; 3.7% were unemployed and 18.1% were not in the labour force (Table 2). MHI scores were on average four points lower in waves with disability compared to waves with no disability (68.5 versus 72.1, Table 2). Both in waves with and without disability, people who were unemployed had the lowest scores. Prior to disability, MHI scores were similar across categories of occupational skill level, but subsequent to disability acquisition, there was a gradient of decreasing mean MHI scores with declining occupational skill level. MHI scores were higher for people who were self-employed and in permanent employment, and lower for those in fixed-term and casual employment, both before and after disability acquisition.

**Table 1:** Sample characteristics at baseline (*n* = 1998)

	*N*	%
Age (years), mean (SD)	42.3	10.2
Sex		
Men	910	45.6
Women	1088	54.5
Country of birth (*n* = 1996)		
Australia	1574	78.9
Other	422	21.1
Education (*n *= 1997)		
Less than secondary	637	31.9
Secondary/certificate/diploma	898	45.0
University education	462	23.1

**Table 2:** Distribution of employment characteristics two waves prior to disability acquisition for those who were employed and mean within-person mental health score in waves reporting no disability and waves reporting disability (*n* = 1998)

	Baseline		Mean MH No disability Mean (SD)	Mean MH Disability Mean (SD)
	*n*	%		
Whole sample	1998	100.0	72.1 (14.8)	68.5 (17.1)
Occupational skill level				
High skill	582	29.1	72.9 (13.8)	70.6 (16.5)
Medium skill	609	30.5	73.3 (14.0)	69.9 (16.8)
Low skill	372	18.6	72.3 (15.5)	67.1 (17.5)
Unemployed	73	3.7	67.1 (15.7)	63.5 (17.1)
Not in the labour force	362	18.1	69.5 (16.4)	65.1 (17.3)
Contract type				
Permanent	937	46.9	73.0 (14.3)	69.6 (17.1)
Fixed term	109	5.5	70.1 (14.6)	66.6 (17.8)
Self-employed	283	14.2	74.6 (13.5)	71.6 (16.1)
Casual	234	11.7	71.9 (14.9)	67.9 (16.4)
Unemployed	73	3.7	67.1 (15.7)	63.5 (17.1)
Not in the labour force	362	18.1	69.5 (16.4)	65.1 (17.3)

### Regression analyses

The overall effect of disability acquisition on mental health was estimated to be more than a four-point decline on the MHI scale (estimated mean difference in MHI between waves with and without disability: − 4.3, 95% CI − 5.0, − 3.5, Table 3). There was strong evidence of effect modification of the relationship between disability acquisition and mental health by occupational skill level (*p* = 0.026) but not contract type (*p* = 0.800).

**Table 3:** Results of the primary analysis: linear fixed-effects regression coefficients for the estimated within-person mean difference in mental health score between waves reporting disability and no disability and interaction terms representing the additional within-person effect of disability on mental health for categories of employment characteristics separately (*n* = 2096, *N* = 16,949)

	Effect of disability		Interaction term		*p* value
	Coeff^a,b^	95% CI	Coeff	95% CI	
Overall effect	− 4.3	− 5.0, − 3.5			
Occupational skill level					
High skill	− 3.0	− 4.2, − 1.7	0		
Medium skill	− 4.0	− 5.2, − 2.8	− 1.0	− 2.7, 0.7	
Low skill	− 6.1	− 7.6, − 4.5	− 3.1	− 5.0, − 1.2	
Not in the labour force	− 4.8	− 6.2, − 3.3	− 1.8	− 3.6, 0.1	
Unemployed	− 4.6	− 8.0, − 1.2	− 1.6	− 5.2, 2.0	*p* = 0.026
Contract type					
Permanent	− 4.0	− 5.0, − 3.0	0		
Fixed term	− 4.2	− 7.3, − 1.1	− 0.2	− 3.4, 2.9	
Self-employed	− 3.6	− 5.4, − 1.8	0.4	− 1.7, 2.4	
Casual	− 5.3	− 7.3, − 3.3	− 1.3	− 3.5, 0.8	
Not in the labour force	− 4.7	− 6.2, − 3.3	− 0.8	− 2.5, 1.0	
Unemployed	− 4.6	− 6.1, − 3.1	− 0.6	− 4.1, 2.9	*p* = 0.800

^a^Results were obtained using multiple imputation using chained equations with 50 imputed data sets

^b^Models were adjusted for age

People in low-skilled jobs experienced on average a six-point decline in MHI when they acquired a disability (− 6.1, 95% CI − 7.6, − 4.5) compared to a four-point decline for people in medium-skilled jobs and a three-point decline for those in high-skilled jobs. People who were unemployed or not in the labour force also experienced large mental health declines of almost five-points on the MHI scale. For contract type, the effects were similar across the categories, with the largest effect for people in casual employment who experienced on average a 5.3-point decline in MHI. The results of the complete case analysis (Table S2 in the Online Resource) and the sensitivity analyses (Table S3 in the Online Resource) were similar to the primary analysis.

## Discussion

The findings of this study suggest that type of occupation prior to disability acquisition influences the magnitude of the effect of disability on mental health, but not contract type. People in low-skilled jobs had more than twice the mental health decline associated with disability acquisition compared to those in high-skilled jobs. People in low-skilled jobs experienced a six-point decline in mental health score, which is considerably larger than the four to five-point difference considered to represent a clinically meaningful difference in mental health [39, 40]. People who were unemployed or not in the labour force prior to disability acquisition also experienced large mental health declines.

This study had a number of strengths. It used longitudinal data from a large nationally representative longitudinal study of Australian households. We used fixed-effects regression, using a within-person analytic design to control for time-invariant individual characteristics. Fixed-effects regression can thus minimise the risk of bias from confounding by unmeasured (or poorly measured) variables that are stable over time.

There were also a number of limitations. There were missing data for 9% of observations in the sample. Examination of the patterns of missingness suggested that there were differences in the observed characteristics of people with missing observations compared to those with complete data, which could have led to selection bias. We used multiple imputation to assess the impact of selection bias from missing data on the results and found very similar results to the complete case analysis, suggesting that missing data are unlikely to have substantially biased the results. There is also the possibility of attrition bias, as we did not impute data for people who were lost to follow-up, however, attrition was low, on average 6% between waves. Another limitation is dependent misclassification bias, which results from the dependency between misclassification of the exposure and outcome. As both disability and mental health were self-reported, it is likely that there was measurement error in the reporting of both measures, and a possibility that the measurement errors were correlated. However, the fixed-effects approach can address this problem to some extent, accounting for any measurement error for an individual that is stable over time, such as negative affectivity. There may have been residual confounding by time-varying confounders. Income, household structure and relationship status are likely to be confounders but also mediators of the association and were therefore not included in the models. However, their inclusion did not affect the effect estimates, therefore residual confounding due to these variables is unlikely. The fixed-effects models assume a contemporaneous effect of disability on mental health that does not change over time. While this is a strong assumption, as for some individuals, the effect may decrease over time, restricting the analysis to two waves before and after disability acquisition did not materially change the findings. Disability acquisition was defined as two consecutive waves of no disability followed immediately by two consecutive waves of disability. This narrow definition may not capture all forms of disability, however, it identifies impairments that are less likely to be transient in presentation. Furthermore, people with severe disabilities are likely to be underrepresented within the sample, particularly those with intellectual or psychological impairments, because they may be less likely to respond to the survey and because the survey samples households from private dwellings only, thereby excluding people with more severe disability who may be living in care facilities. This may lead to an underestimation of the true effect of disability on mental health.

The findings are consistent with previous research which found that the effect of disability on mental health was greater for people who were unemployed [6] and those who experience socioeconomic disadvantage across a number of indicators [5–10]. Our study identified people in low-skilled occupation as a subgroup of people with disabilities who are particularly vulnerable to poor mental health, with some evidence of larger effects than for those who were unemployed or not in the labour force prior to disability.

There are a number of explanations for the gradient in the magnitude of mental health decline according to people’s occupational skill level including economic, psychosocial and other pathways. It may be that people in low-skilled occupations are more likely to lose their job when they acquire a disability, with impacts to income and financial security; identity, status and self-esteem; social contact and social support; and health-related behaviours [41]. Alternatively, people in low-skilled jobs may experience poorer working conditions such as high job demands and low flexibility, which may further contribute to the negative psychosocial impact of disability [42]. It was not possible to investigate the mechanisms by which this effect was operating in this study because the effect modifiers were measured prior to disability acquisition. Future research should aim to disentangle the pathways explaining the effect modification.

Despite the limitations of the study, the robust statistical methods, the consistency of the results with previous research and the magnitude of the effects estimates highlight the importance of people with disabilities’ occupation for their mental health. The findings suggest that interventions on employment characteristics could mitigate the effect of disability on mental health and reduce the impact of structural inequalities on mental health [43]. In addition to provision of high-quality accessible and affordable mental health services for people with disabilities, social interventions that focus on increasing employment rates, improving the sustainability of employment, and providing employment services and education and training opportunities for people who acquire a disability, particularly for people in low-skilled occupations, may improve mental health outcomes.

## Electronic supplementary material

Below is the link to the electronic supplementary material.

Supplementary material 1 (DOCX 32 kb)

## Abbreviations

HILDA: Household, Income and Labour Dynamics in Australia
ICF: International Classification of Functioning, Disability and Health
MHI: Mental health inventory
MI: Multiple imputation
SF-36: Short form 36 health questionnaire
