# Supplementary material for: Does the effect of disability acquisition on mental health differ by employment characteristics? A longitudinal fixed-effects analysis
Source: Soc Psychiatry Psychiatr Epidemiol. 2019 Oct 24;55(8):1031–9. doi: 10.1007/s00127-019-01783-x (PMC7395044; doi:10.1007/s00127-019-01783-x)
Supplement: Supplementary file 1 — Supplementary material 1 (DOCX 32 kb) [file 127_2019_1783_MOESM1_ESM.docx]

Table S1. Distribution of demographic, socio-economic and employment characteristics and mental health for those with complete data and those with missing data

|  | Complete data  n=15,410 | | Missing data  n=1,539 | | P value |
| --- | --- | --- | --- | --- | --- |
|  | n | % | n | % |  |
| **Age (mean, SD)** | 46.4 | 10.1 | 43.3 | 10.2 | <0.001 |
| **Sex** |  |  |  |  |  |
| Men | 6888 | 44.7 | 783 | 50.9 |  |
| Women | 8522 | 55.3 | 756 | 49.1 | <0.001 |
| **Country of birth** |  |  |  |  |  |
| Australia | 12,257 | 79.5 | 1115 | 72.5 |  |
| Other | 3144 | 20.4 | 422 | 27.4 |  |
| Missing | 9 | 0.1 | 2 | 0.1 | <0.001 |
| **Education** |  |  |  |  |  |
| Less than secondary | 4731 | 30.7 | 563 | 36.6 |  |
| Secondary/certificate/diploma | 7144 | 46.4 | 728 | 47.3 |  |
| University education | 3530 | 22.9 | 247 | 16.1 |  |
| Missing | 5 | 0.1 | 1 | 0.1 | <0.001 |
| **Household income quintiles** |  |  |  |  |  |
| Q1 (lowest) | 2175 | 14.1 | 311 | 20.2 |  |
| Q2 | 2611 | 16.9 | 293 | 19.0 |  |
| Q3 | 3149 | 20.4 | 312 | 20.3 |  |
| Q4 | 3476 | 22.6 | 297 | 19.3 |  |
| Q5 (highest) | 3999 | 26.0 | 326 | 21.2 | <0.001 |
| **Relationship status** |  |  |  |  |  |
| In a relationship | 11262 | 73.1 | 1027 | 66.7 |  |
| Not in a relationship | 4148 | 26.9 | 512 | 33.3 | <0.001 |
| **Occupational skill level** |  |  |  |  |  |
| High skill | 4561 | 29.6 | 367 | 23.9 |  |
| Medium skill | 4678 | 30.4 | 411 | 26.7 |  |
| Low skill | 2935 | 19.1 | 398 | 25.9 |  |
| Unemployed | 2706 | 17.6 | 277 | 18.0 |  |
| Not in the labour force | 530 | 3.4 | 86 | 5.6 | <0.001 |
| **Contract type** |  |  |  |  |  |
| Permanent | 7273 | 47.2 | 613 | 39.8 |  |
| Fixed term | 796 | 5.2 | 81 | 5.3 |  |
| Self-employed | 2154 | 14.0 | 227 | 14.8 |  |
| Casual | 1951 | 12.7 | 205 | 13.3 |  |
| Unemployed | 2706 | 17.6 | 277 | 18.0 |  |
| Not in the labour force | 530 | 3.4 | 86 | 5.6 |  |
| Missing | 0 | 0 | 50 | 3.3 | <0.001 |
| **Mental health (mean, SD)** | 70.5 | 18.7 | 64.6 | 20.4 | <0.001 |

Table S2. Results of the complete case analysis: linear fixed-effects regression coefficients and interaction terms for categories of employment characteristics (n=1998)

|  | Effect of disability | | Interaction term | | P value |
| --- | --- | --- | --- | --- | --- |
|  | Coeff^a^ | 95% CI | Coeff | 95% CI |  |
| **Overall effect** | -3.7 | -4.4, -3.0 |  |  |  |
| **Occupational skill level** |  |  |  |  |  |
| High skill | -2.4 | -3.6, -1.2 | 0 |  |  |
| Medium skill | -3.4 | -4.6, -2.2 | -1.0 | -2.6, 0.7 |  |
| Low skill | -5.6 | -7.1, -4.0 | -3.2 | -5.0, -1.3 |  |
| Not in the labour force | -4.5 | -6.0, -3.0 | -2.1 | -3.9, -0.2 |  |
| Unemployed | -3.9 | -7.1, -0.7 | -1.5 | -4.8, 1.9 | p<0.001 |
| **Contract type** |  |  |  |  |  |
| Permanent | -3.4 | -4.4, -2.5 | 0 |  |  |
| Fixed term | -3.1 | -6.0, -0.2 | 0.3 | -2.6, 3.3 |  |
| Self-employed | -3.2 | -4.9, -1.4 | 0.3 | -1.7, 2.3 |  |
| Casual | -4.7 | -6.8, -2.6 | -1.3 | -3.5, 0.9 |  |
| Not in the labour force | -4.5 | -6.0, -3.0 | -1.1 | -2.8, 0.6 |  |
| Unemployed | -3.9 | -7.1, -0.7 | -0.4 | -3.7, 2.8 | p=0.250 |

^a^*models were adjusted for age*

Table S3. Results of the sensitivity analyses: linear fixed-effects regression coefficients and interaction terms for categories of employment variables separately for (1) the analysis removing people with psychological impairments (n=1880, N=15157) and (2) the analysis using the sample restricted to two waves preceding and two waves following disability acquisition (n=2096, N=8384)

|  | Effect of disability | | Interaction term | | P value |
| --- | --- | --- | --- | --- | --- |
|  | Coeff^a,b^ | 95% CI | Coeff | 95% CI |  |
| (1) Psychological impairments removed | | | | | |
| **Overall effect** | -3.7 | (-4.4, -3.0) |  |  |  |
| **Occupational skill level** |  |  |  |  |  |
| High skill | -2.3 | (-3.5, -1.1) |  |  |  |
| Medium skill | -3.6 | (-4.9, -2.4) | -1.4 | (-3.0, 0.3) |  |
| Low skill | -5.5 | (-7.0, -4.0) | -3.2 | (-5.0, -1.4) |  |
| Not in the labour force | -3.9 | (-5.5, -2.3) | -1.6 | (-3.5, 0.3) |  |
| Unemployed | -4.9 | (-8.4, -1.3) | -2.6 | (-6.2, 1.1) | p=0.010 |
| **Contract type** |  |  |  |  |  |
| Permanent | -3.4 | (-4.4, -2.5) |  |  |  |
| Fixed term | -3.2 | (-6.1, -0.3) | 0.2 | (-2.7, 3.2) |  |
| Self-employed | -3.6 | (-5.4, -1.7) | -0.1 | (-2.2, 2.0) |  |
| Casual | -4.4 | (-6.3, -2.5) | -0.9 | (-3.0, 1.1) |  |
| Not in the labour force | -3.9 | (-5.5, -2.3) | -0.4 | (-2.2, 1.4) |  |
| Unemployed | -4.9 | (-6.4, -3.3) | -1.4 | (-5.0, 2.2) | p=0.919 |
| (2) Sample restricted to two waves preceding and two waves following disability acquisition | | | | | |
| **Overall effect** | -2.8 | (-4.1, -1.5) |  |  |  |
| **Occupational skill level** |  |  |  |  |  |
| High skill | -1.5 | (-3.2, 0.2) |  |  |  |
| Medium skill | -2.9 | (-4.5, -1.3) | -1.4 | (-3.1, 0.3) |  |
| Low skill | -4.1 | (-6.0, -2.2) | -2.6 | (-4.6, -0.6) |  |
| Not in the labour force | -3.7 | (-5.7, -1.8) | -2.2 | (-4.2, -0.2) |  |
| Unemployed | -2.0 | (-6.0, 2.0) | -0.5 | (-4.4, 3.5) | p=0.080 |
| **Contract type** |  |  |  |  |  |
| Permanent | -2.6 | (-4.1, -1.2) |  |  |  |
| Fixed term | -3.0 | (-6.4, 0.3) | -0.4 | (-3.8, 3.0) |  |
| Self-employed | -2.0 | (-4.1, 0) | 0.6 | (-1.3, 2.5) |  |
| Casual | -3.4 | (-5.7, -1.2) | -0.8 | (-3.0, 1.4) |  |
| Not in the labour force | -3.7 | (-5.7, -1.8) | -1.1 | (-2.9, 0.7) |  |
| Unemployed | -2.0 | (-3.9, 0) | 0.7 | (-3.2, 4.6) | p=0.732 |

^a^*results were obtained using multiple imputation using chained equations with 50 imputed datasets*

^b^*models were adjusted for age*
